# Curative resolution of chronic thromboembolic pulmonary hypertension with pulmonary thromboendarterectomy in primary antiphospholipid syndrome: A case report

**DOI:** 10.1097/MD.0000000000012710

**Published:** 2018-10-05

**Authors:** Can Li, Jiuliang Zhao, Kun He, Yan Wu, Sheng Liu, Qian Wang, Yan Zhao

**Affiliations:** a Department of Rheumatology; b Department of Medicine, Peking Union Medical College Hospital; c Department of Cardiology; d Department of Cardiac Surgery, Fu Wai Hospital, Chinese Academy of Medical Science, Beijing, China.

**Keywords:** antiphospholipid antibodies, antiphospholipid syndrome, chronic thromboembolic pulmonary hypertension, pulmonary thromboendarterectomy

## Abstract

**Rationale:**

Chronic thromboembolic pulmonary hypertension (CTEPH) is a severe complication of antiphospholipid syndrome (APS). Once diagnosed, the recommendation for the treatment of CTEPH is long-term anticoagulation and pulmonary thromboendarterectomy (PTE). However, cardiac surgeons apply PTE cautiously for these patients, as there is an increased risk of perioperative complications. Here, we present the curative case of a patient with severe APS-associated CTEPH treated with PTE.

**Patient concerns:**

A 29-year-old man presented with chest pain, decreased exercise capacity, dyspnoea, and haemoptysis.

**Diagnoses:**

He was triple positive for antiphospholipid antibodies. Computed tomography pulmonary angiography revealed multiple, recurrent pulmonary embolisms and complete obstruction of the left pulmonary artery. He was diagnosed with APS and CTEPH.

**Interventions:**

After balancing the risk of thrombosis and haemorrhage, the patient underwent PTE.

**Outcomes:**

The patient experienced symptom relief after PTE, and electrocardiography at a six-month follow-up showed a recovery of cardiac structure and pulmonary arterial pressure.

**Lessons:**

After evaluating the thrombosis risk at an experienced treatment centre and the application of standard anticoagulation treatment, PTE may be a curative resolution for APS-associated CTEPH.

## 1 Introduction

Antiphospholipid syndrome (APS) is a disease that manifests clinically as vascular thrombosis (arterial, venous, or small vessel thrombosis) and/or abortion with laboratory results of persistently elevated levels of antiphospholipid antibodies (aPL).^[1]^ Chronic thromboembolic pulmonary hypertension (CTEPH) is a severe complication of patients with APS. According to the previous literature, the 3-year survival rate of patients with CTEPH is only 37% without intervention.^[2]^ The symptoms of CTEPH are not specific, which makes it difficult to diagnose CTEPH at an early stage. Once CTEPH is diagnosed, the recommended treatment is pulmonary thromboendarterectomy (PTE) and life-long anticoagulation.^[3]^ Although PTE is the optimal therapy, patients with APS-associated CTEPH are at risk for the development of complications such as thrombocytopenia ^[4]^ and antiphospholipid syndrome nephropathy,^[5]^ as well as perioperative thrombotic events and bleeding.^[6]^ For this reason, cardiac surgeons apply PTE cautiously for these patients. Here, we present a curative case of severe APS-associated CTEPH treated with PTE. This case shows that PTE is the optimal treatment for patients with CTEPH that has developed from APS. After a full evaluation of the risks of thrombotic events and bleeding and the effective medical treatments that will provide a suitable condition for operation, APS-associated CTEPH should be treated with PTE to improve cardiac function and provide a better prognosis.

## 2 Case report

A 29-year-old man was admitted to the hospital for chest pain, decreased exercise capacity, and hemoptysis. The otherwise healthy patient had developed chest pain in September 2015, with a dull pain in the left shoulder and back and paroxysmal cough. Computed tomography pulmonary angiography (CTPA) revealed a left inferior lobe pulmonary embolism (PE). The patient was prescribed rivaroxaban 20 mg qd, which resolved the pain symptoms.

One month later, he experienced a symptom recurrence and developed shortness of breath after some activities. Laboratory results revealed abnormally high titers of lupus anticoagulant (2.90 with a reference range of ≤1.2), anticardiolipin antibodies (>120 IgG U/mL with a reference range of < 12 IgG U/mL), and anti-beta-2-glycoprotein I (188 RU/mL with a reference range of < 20 RU/mL), indicative of higher thrombosis risk of primary antiphospholipid syndrome (PAPS). Ultrasonic cardiography (UCG) revealed an enlarged right ventricle and atrium, while his pulmonary artery (PA) systolic pressure was 98 mm Hg with an ejection fraction of 63%. He was diagnosed with CTEPH and treatment with hydroxychloroquine (HCQ) 200 mg bid and sildenafil 20 mg tid relieved the symptoms.

Three months later, the patient returned to the hospital because of hemoptysis. A computed tomography scan of the chest showed multiple ill-defined and patchy ground-glass opacities and nodules. Laboratory results showed an NT-proBNP level of 4244 pg/mL.

The patient did not exhibit a rash or experience joint pain, photosensitization, or dryness of the mouth or eyes throughout the disease duration. He had no history of hypertension, diabetes mellitus, hypercholesterolaemia, surgery, malignancy, or other thrombosis risk factors. He had smoked 15 cigarettes per day for many years but had quit 8 months earlier.

**Figure 1:**
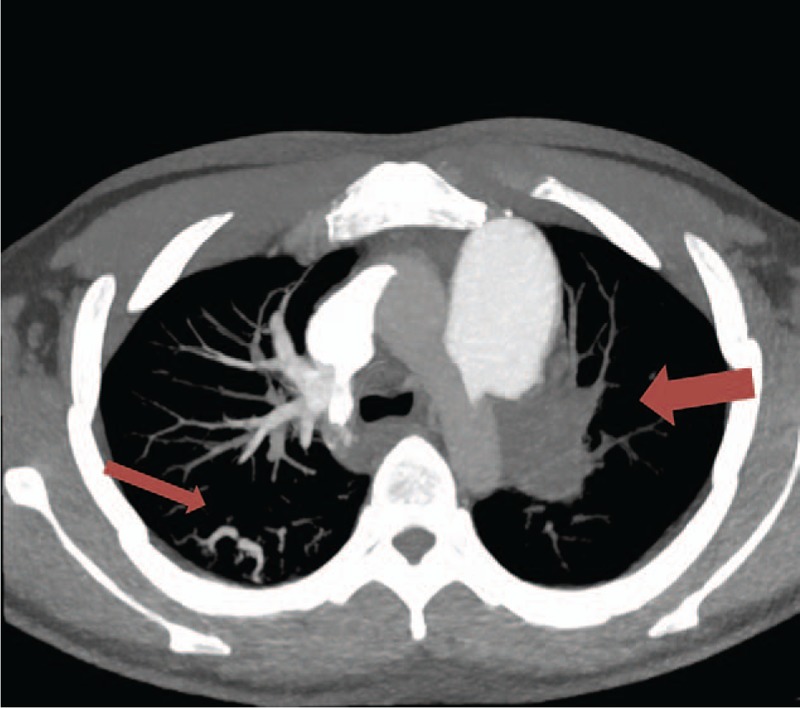
Computed tomography pulmonary angiography revealed multiple pulmonary embolisms, including a complete embolism of the left pulmonary artery (PA) and partial in the branches of the right PA.

During his hospitalization, the anticoagulant therapy was suspended and he was given a continuous intravenous drip of pituitrin. Two weeks later, the hemoptysis was under control. However, CTPA demonstrated multiple PEs, complete obstruction of the left PA, and recurrent thrombosis of the right lung (Fig. 1). The patient's thrombosis was initially treated with low-molecular-weight heparin at 4000 IU qd by hypodermic injection, but he again experienced hemoptysis with ∼ 200 to 300 mL of blood. Bronchial arteriography showed a pulmonary bronchial shunt of the left lung. The hemoptysis was finally successfully treated by bronchial artery embolization. The anticoagulation treatment was continued under close surveillance, and the hemoptysis and dyspnoea did not recur.

**Figure 2:**
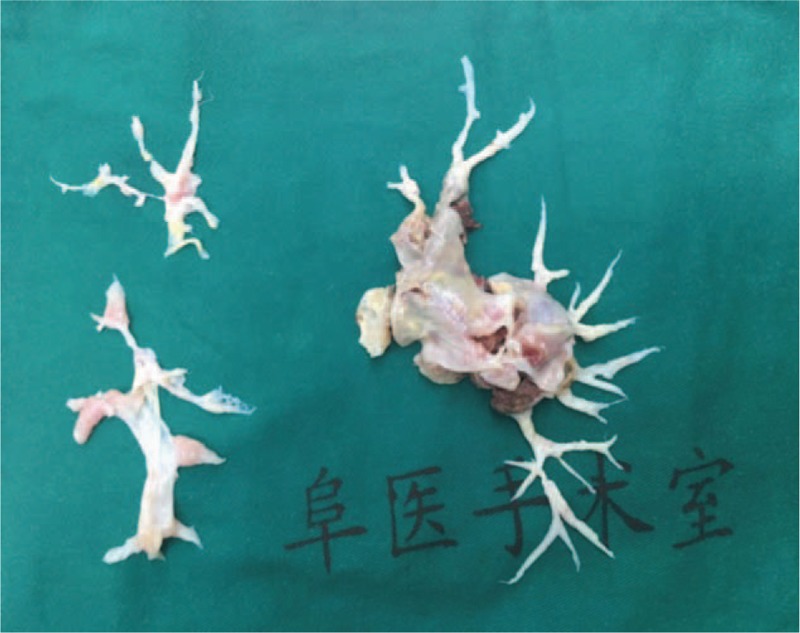
Excised pulmonary artery intima.

Three months later, the patient underwent PTE (Fig. 2). The mean pulmonary arterial pressure (mPAP) was 30 mm Hg determined by right heart catheterization before PTE, and postoperative mPAP was 17 mm Hg. No complications were observed for the patient. After the surgery, the patient reported a markedly increased exercise capacity. Although the patient retained positive aPL titers, UCG revealed that the PA systolic pressure and cardiac structure returned to normal. Life-long warfarin and HCQ 200 mg bid were used to maintain the curative effect. The patient remained well at the 6-month follow-up.

## 3 Discussion

Here, we report a case of APS-associated CTEPH that was curatively treated by PTE. Although the patient was initially treated for an acute PE, subsequent antibody titers and imaging indicated that the patient had PAPS and CTEPH.

CTEPH was defined as a mPAP of ≥25 mm Hg with normal wedge pressure by right heart catheterization, with effective anticoagulation for more than 3 months as well as typical signs on a radionuclide ventilation/perfusion scan and high-quality multidetector CTPA or pulmonary angiography.^[7]^ During the disease course, the patient experienced multiple thrombotic events. Deep vein thrombosis and PE are the most common thrombotic events in patients with APS, with multiple and recurrent thromboses. Indeed, the thrombotic event recurrence rate is nearly 30%.^[8]^ It is reported that 0.57% to 4.7% of cases of acute PE will develop into CTEPH. Common risk factors for CTEPH include unexplained, recurrent, or large clotted PEs, elder age, a chronic inflammatory state, and malignancy.^[2]^ CTEPH pathology may also be associated with high aPL titers,^[9]^ as several cohort studies report aPL-positive titers in ∼15% to 50% of CTEPH patients.^[10,11]^ aPLs may induce CTEPH by inhibiting cascade reactions catalyzed by phospholipids or by promoting microembolization.^[12]^ Indeed, high titers of lupus anticoagulant are associated with microembolization in PAPS, where higher aPL titers correlate with a worse prognosis.^[13]^

Life-long anticoagulation is recommended for CTEPH patients. The patient in the present case was triple-aPL-positive, resulting in a higher risk of thrombosis. Triple aPL positivity is reportedly associated with a high risk for both first and recurrent thrombotic events.^[14]^ The patient, in this case, was previously a heavy smoker and also had an infection, both of which are risk factors for thrombosis.^[15]^ Although rivaroxaban is not reported as inferior to warfarin, it might not be as effective for triple-aPL-positive patients.^[16]^ The patient in the present case was not properly diagnosed at an early stage, and so he missed the optimal therapeutic window for anticoagulation therapy. However, anticoagulants should be used to treat CTEPH with caution. CTEPH is associated with blockage of both the proximal and distal PAs, which increases the blood supply provided by the bronchial artery to the PA and the blood flow volume in the capillary network between these 2 arteries. This increased blood volume makes the capillaries more sensitive to rupture, and so patients with CTEPH should receive anticoagulants under close surveillance.

After the patient's condition was stabilized with medication, he underwent PTE, which was successful. He felt obvious relief of his symptoms after the operation, and UCG showed the recovery of cardiac structure and PA pressure at the 6-month follow-up. From this case, we saw that balancing the risk of thrombosis and hemorrhage can provide a favorable condition for the PTE operation, which is an optimal therapy for APS-associated CTEPH. This result was consistent with previous data.^[6]^

The patient used warfarin and HCQ 200 mg bid after PTE. Since anticoagulation was recommended for all CTEPH patients as a life-long treatment, HCQ has been proven to be effective to prevent postoperative venous thrombotic events.^[17]^ Other target treatments include statin therapy to reduce thrombotic risks and rituximab for refractory thrombosis.

## 4 Conclusion

PTE is a curative option for CTEPH patients and also suitable for those with CTEPH that developed from APS. PTE markedly alleviated the patient's symptoms in this case and restored normal cardiac structure and PA pressure. Thus, after evaluating the thrombosis risk at an experienced treatment centre and applying standard anticoagulation treatment, APS-associated CTEPH may be treated with PTE.

## Author contributions

**Conceptualization:** Jiuliang Zhao, Qian Wang, Yan Zhao.

**Data curation:** Kun He, Yan Wu, Sheng Liu.

**Project administration:** Yan Zhao.

**Resources:** Jiuliang Zhao, Yan Wu, Sheng Liu, Qian Wang.

**Writing – original draft:** Can Li.

**Writing – review & editing:** Jiuliang Zhao.

Can Li: ORCID 0000-0002-0960-9942

## References

[1] Miyakis S Lockshin MD Atsumi T International consensus statement on an update of the classification criteria for definite antiphospholipid syndrome (APS). J Thromb Haemost 2006;4:295–306. 16420554 10.1111/j.1538-7836.2006.01753.x

[2] Delcroix M Kerr K Fedullo P Chronic thromboembolic pulmonary hypertension: epidemiology and risk factors. Ann Am Thorac Soc 2016;13:S201–6. 27571001 10.1513/AnnalsATS.201509-621AS

[3] Kim NH Delcroix M Jenkins DP Chronic thromboembolic pulmonary hypertension. J Am Coll Cardiol 2013;62:D92–9. 24355646 10.1016/j.jacc.2013.10.024

[4] Galli M Finazzi G Barbui T Thrombocytopenia in the antiphospholipid syndrome. Br J Haematol 1996;93:1–5. 10.1046/j.1365-2141.1996.390969.x 8611440

[5] Espinosa G Santos E Cervera R Adrenal involvement in the antiphospholipid syndrome: clinical and immunologic characteristics of 86 patients. Medicine (Baltimore) 2003;82:106–18. 12640187 10.1097/00005792-200303000-00005

[6] Camous J Decrombecque T Louvainquintard V Outcomes of patients with antiphospholipid syndrome after pulmonary endarterectomy. Eur J Cardiothorac Surg 2014;46:116–20. 24362260 10.1093/ejcts/ezt572

[7] Galiè N Humbert M Vachiery JL 2015 ESC/ERS Guidelines for the diagnosis and treatment of pulmonary hypertension. EuroPTEn Heart J 2016;31:1219–63. 10.1016/j.rec.2016.01.002 26837729

[8] Cervera R Piette JC Font J Antiphospholipid syndrome: clinical and immunologic manifestations and patterns of disease expression in a cohort of 1,000 patients. Arthritis Rheum 2002;46:1019–27. 11953980 10.1002/art.10187

[9] Porres-Aguilar M Pena-Ruiz MA Burgos JD Chronic thromboembolic pulmonary hypertension as an uncommon presentation of primary antiphospholipid syndrome. J Natl Med Assoc 2008;100:734–6. 18595578 10.1016/s0027-9684(15)31351-1

[10] Levarge BL Channick RN Chronic thromboembolic pulmonary hypertension: evolution in management. Curr Opin Pulm Med 2014;20:400–8. 25093673 10.1097/MCP.0000000000000088

[11] Wolf M Boyer-Neumann C Parent F Thrombotic risk factors in pulmonary hypertension. Eur Respir J 2000;15:395–9. 10706510 10.1034/j.1399-3003.2000.15b28.x

[12] Zuily S Wahl D Pulmonary hypertension in antiphospholipid syndrome. Curr Rheumatol Rep 2015;17:478. 25604574 10.1007/s11926-014-0478-8

[13] Stojanovich L Kontic M Djokovic A Pulmonary events in antiphospholipid syndrome: influence of antiphospholipid antibody type and levels. Scand J Rheumatol 2012;41:223–6. 22324785 10.3109/03009742.2011.641580

[14] Chayoua W Kelchtermans H Moore GW Identification of high thrombotic risk triple positive antiphospholipid syndrome patients is dependent on anti-cardiolipin and anti-(2glycoprotein I antibody detection assays. J Thromb Haemost 2018;Epub ahead of print. 10.1111/jth.14261 30079628

[15] Erkan D Yazici Y Peterson MG A cross-sectional study of clinical thrombotic risk factors and preventive treatments in antiphospholipid syndrome. Rheumatology (Oxford) 2002;41:924–9. 12154210 10.1093/rheumatology/41.8.924

[16] Dufrost V Risse J Zuily S Direct oral anticoagulants use in antiphospholipid syndrome: are these drugs an effective and safe alternative to warfarin? A systematic review of the literature. Curr Rheumatol Rep 2016;18:74. 27812956 10.1007/s11926-016-0623-7

[17] Dobrowolski C Erkan D Treatment of antiphospholipid syndrome beyond anticoagulation. Clin Immunol 2018;Epub ahead of print. 10.1016/j.clim.2018.03.001 29510235

